# Glycation at the Gate: A Brain Endothelial Glycocalyx Model and Therapeutic Roadmap for Alzheimer’s Disease

**DOI:** 10.3390/biomedicines14081794

**Published:** 2026-08-10

**Authors:** Rawan Tarawneh

**Affiliations:** Department of Neurology, Saint Louis University, 1008 S. Spring Ave, Saint Louis, MO 63110, USA; rawan.tarawneh@health.slu.edu

**Keywords:** brain endothelium, Alzheimer’s disease, glycation, neurodegeneration

## Abstract

While Alzheimer’s disease (AD) is primarily considered a disorder of protein aggregation, converging evidence from clinical, neuropathological, and mechanistic studies strongly supports the notion that brain endothelial dysfunction is a primary and early event in AD pathogenesis. Brain endothelial pathways are among the most differentially expressed in human AD brains. Brain endothelial alterations precede amyloid deposition and cognitive deficits in experimental AD models and closely parallel the degree of neuronal loss in human AD brains. Despite growing evidence to support brain endothelial contributions to neurodegeneration, studies examining the potential of the brain endothelium as a druggable target in AD have been scarce. Further, there has been a relative paucity of validated fluid biomarkers that can reliably measure brain endothelial injury in AD, independently of overt vascular disease or disruption to other cerebrovascular constituents. In this perspective, we propose a brain endothelial glycocalyx-centric model of AD in which brain endothelial dysfunction, driven predominantly by non-enzymatic glycation and carbonyl stress, acts as a key upstream regulator of aberrant protein trafficking, blood–brain barrier instability, and dysregulated neuro-immune cascades. Further, recent evidence suggests the presence of direct interactions of the brain endothelium with key pathways involved in neuronal survival and synaptic signaling, highlighting potential direct contributions of brain endothelial disturbances to cognitive impairment. Within this framework, we identify several brain endothelial axes, including reduction in carbonyl stress, improved glycation-dependent signaling, attenuation of advanced glycation end-product (AGE)-mediated toxicity, and enhanced endothelial glycocalyx stability and resilience as potential therapeutic approaches in AD. Modulating brain endothelial glycation has potential as a novel therapeutic strategy in AD which may complement other disease-modifying treatments, particularly in the earliest preclinical stages. In conclusion, this framework positions the brain endothelium as a mechanistic hub linking metabolic stress to aberrant protein aggregation and neurodegeneration in AD with potential therapeutic implications in AD and other neurodegenerative disorders.

## 1. Introduction

### Alzheimer’s Disease as a Brain Endotheliopathy

Converging evidence from clinical, genetic, neuropathological, and experimental studies supports an important role for brain endothelial dysfunction as an early and primary process in Alzheimer’s disease (AD) pathogenesis [1,2,3,4,5,6]. Brain endothelial dysfunction, characterized by ultrastructural changes such as increased pinocytotic vesicles, basement membrane thickening, and focal endothelial necrosis is a prevalent pathology which is observed in most (>90%) AD brains, including those with no or only minimal overt vascular pathology (i.e., arteriolosclerosis or atherosclerosis) [2,7,8]. Brain endothelial pathways are among the most differentially expressed in human AD brains [9]; most of the top AD risk genes are expressed in vascular cell types and many are enriched in the brain endothelium [10]. Consistent with these observations, using cerebrospinal fluid (CSF) proteomics studies across several large longitudinal AD cohorts, we have found that brain endothelial proteins represent a significant proportion of differentially expressed proteins in preclinical AD, compared to controls, and are strongly represented as drivers of functional modules associated with AD pathology [11]. Our data-driven disease models also support the notion that brain endothelial proteins are altered very early in AD pathogenesis in temporal proximity to the onset of amyloid deposition, which begins at least a decade prior to symptom onset, and that brain endothelial alterations are closely associated with the progression of amyloid and tau pathologies and predict trajectories of future cognitive decline even in the earliest preclinical stages [12].

## 2. A Proposed Brain-Endothelial Glycocalyx Model of AD

### 2.1. The Endothelial Glycocalyx: A Gate to the Brain

The brain endothelial glycocalyx is a hydrated, negatively charged layer which is rich in heparan sulfate proteoglycans (HSPGs) and lines the luminal surface of the brain endothelium [13]. Beyond serving as a physical and electrostatic barrier to maintain blood–brain barrier (BBB) integrity, it orchestrates growth-factor signaling, receptor- and caveolae-mediated transport, and lipid processing, thereby preserving cerebrovascular metabolic homeostasis and restricting aberrant protein trafficking and aggregation [13,14]. The glycocalyx also exerts immunomodulatory effects by regulating adhesion molecules and coordinating bidirectional communication with microglia, astrocytes, and pericytes [14]. Importantly, the brain endothelial glycocalyx directly influences neuronal and synaptic function by regulating vascular signaling, trophic factor availability, and activity-dependent neurovascular coupling [13,14].

When examining endothelial contributions to AD pathogenesis, protein constituents of the brain endothelial glycocalyx are of particular interest [13]. We have recently shown that upregulation of the brain endothelial glycocalyx protein, syndecan-4 (SDC4), reflected by elevated CSF SDC4 levels, begins very early in preclinical AD, near the onset of amyloid pathology, increases more robustly following the point of tau positivity, and strongly predicts cognitive (i.e., global and domain-specific cognition) and pathological (i.e., brain amyloid and tau aggregation) disease trajectories over a mean follow-up of 8 years in preclinical AD [12]. Further, elevated CSF SDC4 levels predicted our disease models, which depict the sequential progression of individuals through the different clinico-pathological stages of AD over a granular estimate of disease progression measured using pseudo-time, to a potentially better extent than other emerging AD biomarkers that reflect other AD pathologies such as neuronal/synaptic injury and microglial or astrocytic dysregulation [12]. These novel findings build on a growing body of evidence that supports important contributions of the brain endothelial glycocalyx to AD onset and progression, including abnormal protein aggregation and impaired synaptic plasticity, culminating in memory loss and the onset of cognitive impairment [12,13,15].

### 2.2. Brain Endothelial Glycation in AD

A substantial body of translational and mechanistic evidence implicates non-enzymatic glycation as a key driver of brain endothelial dysfunction in AD [16,17]. Non-enzymatic glycation occurs when sugars or reactive dicarbonyls, mainly methylglyoxal (MG) and glyoxal, react with lysine/arginine side chains of endoluminal proteins to form early adducts that rearrange into advanced glycation end-products (AGEs), including carboxymethyl-lysine (CML), carboxyethyl-lysine (CEL), and MG-derived hydroimidazolones (MG-H1) [17]. Aging, glycemic variability, oxidative stress, and impaired detox capacity collectively result in the accumulation of AGEs on the luminal brain endothelial glycocalyx which can alter BBB mechanics or signaling and influence long-lived (e.g., collagens and laminins) or circulating (e.g., albumin, fibrinogen, and apolipoprotein E [ApoE]) proteins [17,18]. Consistent with these observations, neuropathological studies of human AD brains demonstrate increased AGE burden and reduced glyoxalase-1 (GLO1)-mediated detoxification capacity within the cerebral microvasculature, supporting a disease-relevant failure of endothelial carbonyl stress defenses early in the disease process [19,20].

While abnormal non-enzymatic glycation affects multiple proteins in the AD brain, brain endothelial glycocalyx components represent particularly vulnerable and functionally relevant targets [13,15] due to their chemical composition, luminal exposure, and central role in regulating protein trafficking at the blood-brain interface. Integrative and high-resolution structural glycoproteomic studies support the notion that widespread remodeling of protein glycosylation and conformation is an important early molecular feature of AD [21]. Alterations in N- and O-linked glycosylation affect proteins enriched at the neurovascular interface (e.g., apolipoproteins, adhesion molecules, extracellular matrix components, and plasma-derived carriers), many of which interact directly with, or are embedded within, the brain endothelial glycocalyx [15,21]. A marked loss of mucin-domain O-glycosylated proteins, resulting in destabilization of the luminal glycocalyx, and coordinated dysregulation of glycosylation pathways in brain endothelial cells have been reported with aging and neurodegeneration [15].

Transcriptomic profiling studies show that brain endothelial cells are molecularly heterogeneous and regionally specialized, with distinct gene expression patterns along the arteriovenous axis that shift with aging and neurodegeneration, including AD [22,23]. In mouse and human aging brains, zonation-dependent transcriptomic changes are observed across endothelial subtypes, including alterations in pathways regulating glucose/energy metabolism, immune signaling, and key BBB regulators such as MFSD2A, SLC2A1 (glucose transporter type 1 [GLUT1]), and tight junction components [24,25]. These changes overlap significantly with AD genetic risk loci and are functionally relevant to vascular regulation and protein transport [23,25,26]. Importantly, endothelial subtype-specific transcriptional alterations suggest that susceptibility to metabolic stress, glycan disruption, and protein handling across the cerebrovascular network may vary by vascular segment, providing a plausible mechanistic basis for regional vulnerability to glycoproteome remodeling and subsequent carbonyl modification at the brain endothelial interface [22,23,24,25,26].

Interestingly, high-resolution glycoproteomic studies suggest that site-specific remodeling of glycan occupancy and structure in AD, including glycan truncation, reduced terminal sialylation, and altered branching on extracellular and luminal-facing proteins, occurs independently of corresponding global changes in protein abundance [21]. Specifically, large-scale glycoproteomic studies and human brain glycoform-network analyses demonstrate coordinated, site-specific alterations in N- and O-linked glycoforms across proteins involved in lipid transport, extracellular matrix organization, immune signaling, and vascular interactions, suggesting systematic rewiring of glycosylation rather than random stochastic effects [27,28,29,30,31]. Region-specific N-glycome profiling in human AD brains reveals spatially patterned losses of complex and sialylated N-glycans across vulnerable cortical and hippocampal regions, which further supports the notion that glycan destabilization is locally regulated and aligned with regional susceptibility rather than a nonspecific downstream consequence of neurodegeneration [32,33].

### 2.3. Brain Endothelial Glycation as a Primer to AD Pathology

Brain endothelial glycation is mechanistically linked to abnormal protein aggregation through impaired clearance, altered trafficking, and enhanced nucleation of aggregation-prone proteins at the BBB [34,35,36]. Glycation creates a pro-aggregation microenvironment by generating nucleation sites along the luminal endothelial surface, upregulating receptors for advanced glycation end-products (RAGEs), and impairing low-density lipoprotein receptor-related protein-1 (LRP1)-mediated efflux, thereby favoring the influx and vascular retention of amyloid-β (Aβ), tau, and α-synuclein [34,35,36]. Concurrent glycocalyx disruption exposes AGE-modified surfaces that promote oligomer seeding and fibrillization, while AGE-mediated cross-linking of basement membrane and glycocalyx components stiffens the perivascular matrix and impairs glymphatic and periarterial drainage [35,36,37,38]. These structural and signaling changes disrupt astrocytic end-foot integrity, reducing interstitial fluid exchange and protein clearance, while increased endothelial permeability and matrix stiffness further promote interstitial protein crowding [35,36,39]. Beyond the BBB, glycation directly modifies key AD-related proteins, including Aβ, amyloid precursor protein (APP), tau, and α-synuclein, through AGE adduct formation on lysine and arginine residues, increasing their hydrophobicity, and accelerating their β-sheet assembly and oligomer formation [37,40,41,42]. Glycation weakens tau-microtubule binding and may interfere with protective O-GlcNAcylation, enabling kinase-driven hyperphosphorylation [41]. Glycation of α-synuclein disrupts its membrane interactions and proteostasis [42]. Together, these effects promote Aβ, tau, and α-synuclein aggregation.

Brain endothelial glycation also alters glial function. AGE accumulation and endothelial AGE-RAGE signaling increase oxidative and inflammatory stress at the neurovascular interface, shifting microglia towards a pro-inflammatory, clearance-deficient state [43]. Glycation of endothelial-derived ligands, apolipoproteins, and extracellular matrix components may alter microglial receptor engagement, including triggering-receptor expressed on myeloid cells-2 [TREM2]-dependent sensing of lipids and aggregated proteins, thereby impairing microglial clustering and amyloid clearance [44,45]. In astrocytes, chronic AGE-RAGE signaling, serum protein exposure, and redox imbalance may impair glymphatic support, promote reactive astrocytosis and cytoskeletal remodeling, and disrupt end-foot domains that regulate glutamate, potassium, and water homeostasis [39,46]. Collectively, brain endothelial glycation may propagate carbonyl stress and proteostatic dysfunction into glial compartments, coupling endothelial injury to neuroimmune dysregulation, protein aggregation, and neurodegeneration [39,43,44,45,46,47].

In summary, these endothelial, molecular, and glial mechanisms position non-enzymatic glycation as a potential driver of multi-protein aggregation and impaired protein clearance in AD.

### 2.4. Direct Endothelial-Neuronal-Synaptic Cross-Talk

Neuropathological and mechanistic studies suggest close associations between brain endothelial injury and neuronal or synaptic dysfunction in AD [48,49,50,51]. Brain endothelial injury is frequently observed near dystrophic neurites, displays regional and laminar patterns that closely parallel those of neuronal loss, and is mechanistically linked to impaired synaptic plasticity in animal models of AD [1,3,50,52]. Consistent with this notion, we have recently shown strong functional associations of brain endothelial proteins (e.g., cadherin-5; vascular endothelial cadherin [VE-cadherin]) with proteins involved in synaptic plasticity, long-term potentiation (LTP), and memory functions (e.g., semaphorins) [51].

Brain endothelial glycation is associated with impaired synaptic plasticity and axonal growth through direct and indirect mechanisms. Glycation-induced remodeling of the endothelial glycocalyx perturbs endothelial-glial signaling, leading to astrocytic and microglial dysregulation and impaired activity-dependent synaptic potentiation [46,47,53]. Endothelial exposure of serum proteins engages synapse-suppressive pathways, including albumin-driven astrocytic transforming growth factor (TGF)-β signaling and fibrinogen-mediated microglial activation, while associated neuroinflammatory responses promote complement-mediated synaptic tagging and microglia-mediated synaptic and dendritic spine elimination [46,47,54]. Endothelial AGE-RAGE signaling also propagates oxidative and inflammatory stress to neurons, dampening N-methyl-D-aspartate receptor (NMDAR)-dependent plasticity and brain-derived neurotrophic factor (BDNF)-tropomyosin receptor kinase B (TrkB) pathways required for spine maintenance, axonal extension, and neuronal survival [34,49]. Further, brain endothelial glycation disrupts neurovascular coupling by reducing nitric oxide (NO) bioavailability and destabilizing pericyte-mediated capillary responses, producing activity-energy mismatches that limit synaptic plasticity and axonal remodeling [49,55]. AGE cross-linking and protease imbalance can remodel the synaptic extracellular matrix, further contributing to synaptic loss [47,54].

The brain endothelium also engages in direct angiocrine and neuromodulatory signaling that influences synaptic structure and plasticity; glycation-induced disruption of these pathways may further contribute to synaptic failure in AD [56,57,58]. Endothelial-derived BDNF supports neuronal survival, dendritic spine stability, and hippocampal synaptic plasticity, while other endothelial signals (e.g., Wnt ligands, semaphorins, NO, and extracellular vesicles carrying proteins, lipids, and regulatory ribonucleic acids [RNAs]) regulate neuronal survival, axonal growth, synapse formation, and activity-dependent plasticity [52,53,56,57,58]. Glycocalyx remodeling can alter endothelial growth-factor presentation, negatively influencing synaptic repair and resilience. Convergence with vascular endothelial growth factor (VEGF)–VEGF receptor-2 (VEGFR2)–neuropilin-1 (NRP1) permeability pathways provides a plausible mechanistic link between brain endothelial glycation and maladaptive neurovascular signaling [52,59]. By enhancing VEGF bioavailability and altering receptor clustering, glycation-induced glycocalyx disruption may promote exaggerated VEGFR2 activation, Src-mediated VE-cadherin phosphorylation, junctional disassembly, and BBB disruption, while NRP1 co-activation further amplifies cytoskeletal remodeling and increased permeability [52,59,60]. Interestingly, several signaling systems that coordinate axonal guidance and vascular patterning are shared between endothelial and neuronal cells, including semaphorin-NRP1, ephrin-Eph receptor, and netrin signaling pathways [52,53,61,62]. These pathways coordinate vascular branching, endothelial cell migration, and vessel stabilization with axonal navigation, synaptic organization, and neuronal connectivity. For example, NRP1 integrates semaphorin and VEGF signaling to coordinate axonal guidance with angiogenesis, while ephrin-Eph interactions regulate synaptic structure and vascular stabilization [52,61].

Conversely, neuronal and synaptic activity regulates cerebral endothelial behavior through glutamate-mediated neurovascular coupling, neuronal NO signaling, activity-dependent release of vascular growth factors that modulate endothelial metabolism, vascular remodeling and endothelial transcriptional responses that support circuit plasticity [49,63,64,65]. In summary, this framework supports the concept that direct, bidirectional endothelial-neuronal and endothelial-synaptic signaling pathways are structurally and functionally disrupted by abnormal brain endothelial glycation, positioning the brain endothelium as an active regulator of neuronal survival and synaptic integrity in AD.

**Figure 1:**
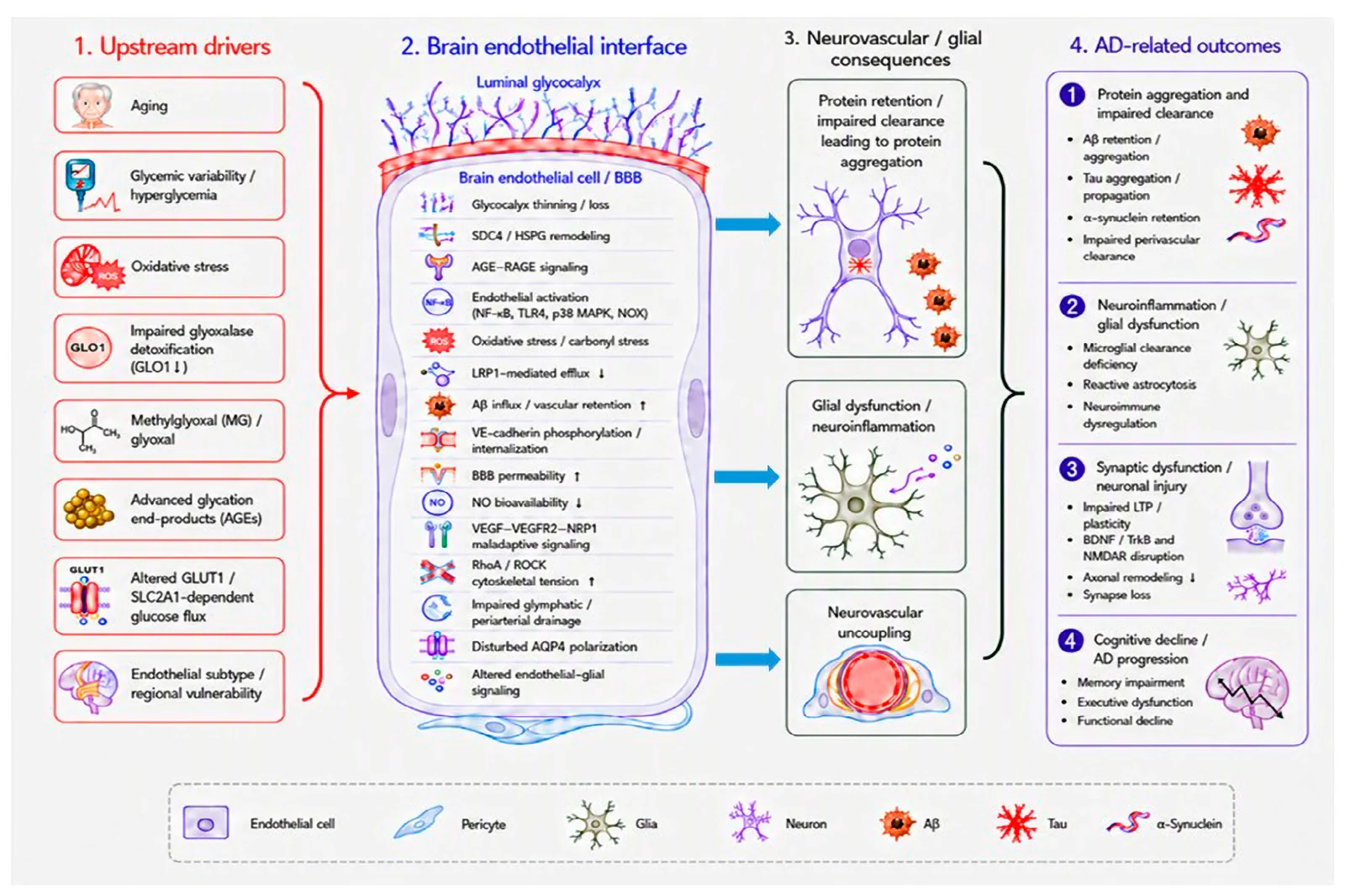
A schematic illustration of the mechanisms by which disruption to the brain endothelial glycocalyx potentially contributes to AD pathogenesis. Abbreviations: Aβ, amyloid-β; AD, Alzheimer’s disease; AGEs, advanced glycation end-products; AQP4, aquaporin-4; BBB, blood–brain barrier; BDNF, brain-derived neurotrophic factor; GLO1, glyoxalase 1; GLUT1, glucose transporter type 1; HSPG, heparan sulfate proteoglycan; LRP1, low-density lipoprotein receptor-related protein 1; LTP, long-term potentiation; MAPK, mitogen-activated protein kinase; MG, methylglyoxal; NF-κB, nuclear factor κB; NO, nitric oxide; NOX, nicotinamide adenine dinucleotide phosphate oxidase; NMDAR, N-methyl-D-aspartate receptor; NRP1, neuropilin-1; RAGE, receptor for advanced glycation end-products; RhoA, Ras homolog family member A; ROCK, Rho-associated coiled-coil-containing protein kinase; ROS, reactive oxygen species; SDC4, syndecan-4; SLC2A1, solute carrier family 2 member 1; TLR4, Toll-like receptor 4; TrkB, tropomyosin receptor kinase B; VE-cadherin, vascular endothelial cadherin; VEGF, vascular endothelial growth factor; VEGFR2, vascular endothelial growth factor receptor 2. This figure was generated and iteratively edited with assistance from ChatGPT (GPT-5.5 Thinking, OpenAI) using scientific content, concepts, and revision instructions provided by the author. The author reviewed the final figure and verified its scientific accuracy.

Figure 1 summarizes the main mechanisms by which disruption to the brain endothelial glycocalyx potentially contributes to AD pathogenesis including protein aggregation, glial, neuronal, and synaptic dysfunction, and cognitive decline.

## 3. A Proposed Brain Endothelial Glycocalyx Therapeutic Roadmap for AD

### Targeting Brain Endothelial Glycation in AD

Brain endothelial glycation represents a convergent therapeutic node linking carbonyl stress, AGE-RAGE signaling, glycocalyx injury, BBB dysfunction, impaired protein clearance, protein aggregation, and neurodegeneration. The following sections outline complementary intervention axes that may serve as potential novel therapeutic approaches in AD.

- Reducing Carbonyl Stress

Given the strong mechanistic link between brain endothelial carbonyl stress and AD pathogenesis, repurposed small molecules that lower reactive dicarbonyls (e.g., MG) or boost endogenous detoxification (e.g., nuclear factor erythroid 2–related factor 2 [Nrf2]-GLO1 axis) represent promising therapeutic candidates to reduce brain endothelial AGE formation. MG is increasingly recognized as a vascular-relevant glycating stressor in AD, with substantial evidence that it impairs brain endothelial function and promotes carbonyl-derived protein modification [16,17,18]. Benfotiamine is a lipid-soluble thiamine derivative which shifts glucose metabolism away from MG-generating intermediates via transketolase-dependent pathways [66]. In a randomized placebo-controlled 12-month trial involving individuals with mild cognitive impairment (MCI) and mild AD dementia, benfotiamine was safe and well tolerated and was associated with significantly less worsening on the Clinical Dementia Rating (CDR) scale, reduced AGE accumulation, and trends towards slower cognitive decline on the Alzheimer’s Disease Assessment Scale-Cognitive subscale (ADAS-Cog), compared with placebo, with stronger effects observed among *APOE4* non-carriers [67]. Building upon these findings, a larger multicenter randomized Phase 2A/2B trial (BenfoTeam) is currently evaluating the safety and efficacy of benfotiamine in early AD [68].

Metformin, a widely prescribed insulin-sensitizing biguanide, represents another promising strategy to attenuate endothelial carbonyl stress through adenosine monophosphate (AMP)-activated protein kinase (AMPK) activation, improved glucose handling, and direct scavenging of reactive dicarbonyls such as MG [69,70]. Given its extensive clinical safety record, it will be of interest to determine whether metformin modulates biomarkers of brain endothelial glycation and injury in AD. Ongoing secondary prevention studies, including the Metformin in Alzheimer’s Dementia Prevention (MAP) trial, will provide important insight into potential metformin effects on AD-related pathways [71].

Complementary “detox amplification” approaches focus on Nrf2-dependent upregulation of GLO1, a central MG defense system. Preclinical studies demonstrate direct transcriptional control of GLO1 by Nrf2, with evidence that Nrf2 activation (e.g., using sulforaphane) enhances GLO1 expression/activity and improves MG-related metabolic stress signaling [72,73]. Clinically used Nrf2 activators with human safety footprints, such as fumarates used in multiple sclerosis, are also of particular interest given emerging evidence that dimethyl fumarate may attenuate carbonyl stress through Nrf2-linked antioxidant and cytoprotective pathways in neuronal systems, providing a translational basis for testing Nrf2-GLO1 engagement as a potential therapeutic strategy in AD [74]. Notably, potential benefits of dimethyl fumarate on cognitive outcomes and slowing clinical disease progression are currently being evaluated in a randomized clinical trial in patients with MCI and AD dementia (ClinicalTrials.gov NCT06850597). Additional carbonyl/AGE-lowering candidates include pyridoxamine, which reduced MG and AGE markers and lowered soluble endothelial activation markers (i.e., soluble vascular cell adhesion molecule-1 [sVCAM-1] and soluble intercellular adhesion molecule-1 [sICAM-1]) in metabolically at-risk individuals in an 8-week, randomized, double-blind, placebo-controlled trial [75]. Findings from this study support target engagement on dicarbonyl stress biology despite limited effects on short-term functional vascular endpoints, such as flow-mediated dilation or arterial stiffness [75,76]. Finally, endogenous dipeptides such as carnosine and anserine offer a complementary scavenger-based strategy to reduce pathologic brain endothelial glycation. Anti-glycation and neuroprotective effects of these agents have been observed in experimental studies [77] as well as possible cognitive benefits in *APOE4* carriers with MCI [78].

Glucose transporters provide an additional mechanistic link between brain metabolic stress and endothelial glycation. In the brain endothelium, GLUT1 (SLC2A1) is a major regulator of glucose transport across the BBB and therefore influences local substrate availability for glycolysis and downstream MG generation [17,79]. Dysregulation of endothelial glucose transport, whether through altered GLUT1 expression, function, or regional endothelial subtype vulnerability, may promote localized glycolytic overflow and carbonyl stress even in the absence of overt systemic diabetes [17,22,24,25,79]. Thus, GLUT1-dependent glucose flux may influence the spatial and temporal vulnerability of the brain endothelial interface to AGE formation and glycocalyx injury. Strategies that normalize GLUT1-dependent glucose flux, glycemic variability, and downstream carbonyl stress [17,24,25,79], rather than directly inhibiting GLUT1 at the BBB, may offer therapeutic benefits in AD, particularly given evidence that GLUT1 reduction exacerbates AD-related vasculo-neuronal dysfunction and degeneration [80].

In silico network prioritization of MG metabolism and endothelial carbonyl stress signaling identifies several potential complementary therapeutic nodes beyond the canonical Nrf2-GLO1 axis; however, many of these targets remain under-investigated as AD therapeutic strategies, and AD-specific experimental or clinical evidence remains limited. These include enzymes involved in downstream glyoxal detoxification such as glyoxalase-2 (HAGH), members of the aldo-keto reductase family (e.g., AKR1B1/AKR1B10), and aldehyde dehydrogenases (e.g., ALDH2), which collectively contribute to the clearance of reactive carbonyl intermediates and AGE precursors [17]. Pharmacologic activation of mitochondrial ALDH2, for example with small-molecule activators such as Alda-1, has shown vascular-protective effects in experimental systems and may represent a translational strategy to enhance endothelial carbonyl detoxification [81]. Network analyses also highlight DJ-1 (PARK7) as a protective deglycase that repairs early glycation adducts and may buffer MG-induced protein damage [82]. Upstream metabolic regulators that limit MG generation, including transketolase-dependent shunting of glycolytic intermediates [66], pentose phosphate pathway enzymes such as glucose-6-phosphate dehydrogenase (G6PD) that sustain nicotinamide adenine dinucleotide phosphate (NADPH) and glutathione pools, and AMPK-linked metabolic stress pathways may similarly attenuate endothelial carbonyl burden [17]. Because MG detoxification through the GLO1/GLO2 glyoxalase system requires an available pool of reduced glutathione, interventions that preserve glutathione availability may further augment endogenous carbonyl defense mechanisms [83]. Together, these targets define a broader therapeutic landscape in which interventions could simultaneously reduce MG production, enhance detoxification capacity, and preserve redox homeostasis, thereby mitigating glycation-driven brain endothelial dysfunction in AD [17,66] (Table 1).

B. Targeting the AGE-RAGE pathway

A second downstream therapeutic axis targets the AGE-RAGE amplification node which couples glycated ligands to altered endothelial signaling and AD-relevant protein trafficking. RAGE, a multiligand pattern-recognition receptor highly expressed on brain endothelial cells, binds AGEs and related ligands (e.g., high-mobility group box 1 [HMGB1] and S100 proteins) stimulating intracellular signaling pathways involving diaphanous-related formin 1 (DIAPH1), nuclear factor κB (NF-κB), and p38 mitogen-activated protein kinase (MAPK), which promote oxidative stress and endothelial activation [84]. In the brain endothelium, RAGE signaling also regulates vascular protein trafficking and mediates the influx of circulating Aβ into the brain, providing a clear mechanistic link between systemic metabolic stress and brain amyloid pathology [34]. The AGE-RAGE pathway functions as a signaling hub integrating metabolic stress, innate immunity, oxidative injury, and amyloid biology. RAGE activation amplifies inflammatory signaling through innate immune receptors such as toll-like receptor 4 (TLR4), enhances NADPH oxidase (NOX)-mediated oxidative stress, reinforces feed-forward endothelial activation, and is associated with increased β-secretase (BACE1) expression and amyloidogenic APP processing, thereby linking endothelial stress to amyloid production [84,85].

The small-molecule high-affinity RAGE inhibitor, FPS-ZM1, blocks Aβ-RAGE interactions and has been shown to reduce RAGE-mediated Aβ influx, lower brain Aβ levels and improve cognition in AD models [86]. This has led to clinical development of the oral RAGE antagonist, azeliragon (**TTP488/PF-04494700**), which has been associated with potential cognitive benefits in early-stage clinical studies; however, late-stage development has been challenged by tolerability concerns and disappointing results of a Phase 3 trial [87]. Therefore, there is a need to identify new RAGE targets that extend beyond direct receptor antagonism.

A potential therapeutic strategy involves increasing soluble RAGE (sRAGE) to function as a decoy for circulating glycated ligands and Aβ, thereby limiting endothelial RAGE activation without direct receptor blockade [88,89]. sRAGE can sequester AGEs, HMGB1, and S100 proteins in the circulation, effectively buffering ligand availability and attenuating inflammatory signaling at the BBB. This approach may offer improved tolerability while indirectly targeting the AGE-RAGE amplification loop at the brain endothelial interface. Beyond direct receptor antagonism, future therapeutic strategies could also target RAGE gene (i.e., *AGER*) transcription and endothelial RAGE expression, thereby suppressing activation of the AGE-RAGE axis upstream of ligand-dependent inflammatory and oxidative stress signaling. Therapeutic modulation of the AGE-RAGE axis may exert disease-modifying effects, not only by limiting ligand-receptor signaling, but also by disrupting downstream inflammatory and amyloidogenic feedback circuits.

While strategies that reduce carbonyl stress or AGE-RAGE activation are likely to be most effective if administered early, established AGE crosslinks on extracellular matrix and glycocalyx-associated proteins may persist and sustain endothelial stiffness and dysfunction. In this context, AGE crosslink-breaking compounds, such as alagebrium (**ALT-711**) which improves vascular compliance and endothelial function in the setting of vascular aging and cardiometabolic disorders, represent a complementary approach to reverse pre-existing brain endothelial AGE burden [38]. Additional endogenous protein repair pathways, including fructosamine-3-kinase (FN3K), may limit AGE accumulation by reversing early glycation adducts before progression to irreversible AGE modifications [90].

In silico network prioritization of the RAGE signaling interactome highlights several additional potential therapeutic nodes, including the intracellular adaptor DIAPH1, RAGE ligands such as HMGB1 and S100 family proteins, cooperating innate immune receptors such as TLR4, and downstream inflammatory and oxidative stress pathways (e.g., NF-κB, p38 MAPK, and NOX), which represent key points of ligand amplification, signal propagation, and amyloidogenic feed-forward signaling, and therefore, may serve as complementary targets to attenuate RAGE-driven brain endothelial dysfunction in AD (Table 1). As evidence for several of these targets in AD remains indirect, further studies are warranted to define their therapeutic relevance and determine whether modulation of these pathways may support disease-modifying strategies in AD.

C. Preserving the Brain Endothelial Glycocalyx

Preservation and stabilization of the brain endothelial glycocalyx offers a distinct potential therapeutic axis for reducing abnormal protein aggregation and protecting BBB integrity under metabolic or carbonyl stress. In this context, membrane-bound proteoglycans (e.g., syndecans and glypicans) and heparan sulfate glycosaminoglycan chains emerge as potential therapeutic targets [12,13,14,15]. SDC4 is a transmembrane HSPG which is upregulated in AD brains and has recently been identified as a potential CSF marker of brain endothelial dysfunction in early preclinical AD [12]. Mechanistically, SDC4/HSPG dysregulation promotes the synthesis, nucleation, aggregation, and internalization of amyloid and tau, impairs their clearance, and has been associated with the increased intercellular spread of tau [12,91]. Secondary effects of SDC4/HSPG remodeling may also converge with barrier-destabilizing pathways, including VEGF-linked VE-cadherin internalization, Ras homolog family member A (RhoA)/Rho-associated coiled-coil-containing protein kinase (ROCK)-dependent cytoskeletal tension, and increased BBB permeability [12,15,60]. Thus, therapeutic modulation of SDC4-linked heparan sulfate remodeling may represent a plausible upstream approach to limit Aβ and tau aggregation and potentially stabilize the BBB.

Intraluminal shedding of proteoglycan ectodomains, driven in part by metalloproteinases such as matrix metalloproteinase 9 (MMP9) and a disintegrin and metalloproteinase domain-containing protein 17 (ADAM17), and degradation of heparan sulfate chains by heparanase can further destabilize glycocalyx structures [92]. Consequently, limiting secondary proteoglycan shedding through inhibition of metalloproteinases, together with preservation of heparan sulfate integrity via heparanase inhibition, represent plausible strategies to stabilize the luminal endothelial interface following glycocalyx injury [92].

In silico network prioritization of glycocalyx regulatory pathways highlights several potential complementary therapeutic nodes (Table 2), including the Angiopoietin-1 (Ang1)/Tie2 axis that supports endothelial quiescence and glycocalyx recovery [93,94]. These pathways promote junctional stability following inflammatory or metabolic injury by reinforcing VE-cadherin retention and cortical actin organization. Therefore, restoration of Ang1/Tie2 axis, through Tie2 activation or vascular endothelial protein tyrosine phosphatase (VE-PTP) inhibition, may offer therapeutic benefits in AD [93,94]. Sphingosine-1-phosphate receptor 1 (S1PR1) signaling may represent another complementary barrier-stabilizing pathway, as it supports endothelial junctional integrity, cortical actin organization, and suppression of RhoA/ROCK-dependent contractility, thereby potentially preserving BBB and glycocalyx stability under inflammatory or carbonyl stress [95]. Pharmacologic modulation of this downstream cytoskeletal axis, including ROCK inhibition with agents such as fasudil or ripasudil, may provide an additional strategy to reduce endothelial contractility, stabilize VE-cadherin-dependent junctions, and protect BBB integrity in AD [95]. Enzymes involved in heparan sulfate biosynthesis (e.g., exostosin glycosyltransferases [EXT1/EXT2], N-deacetylase/N-sulfotransferase 1 [NDST1], and heparan sulfate 6-O-sulfotransferase 1 [HS6ST1]), which modify the structure and sulfation patterns of proteoglycans within the endothelial glycocalyx and vascular basement membrane (e.g., perlecan [HSPG2] and agrin [AGRN]), also contribute to glycocalyx stability and barrier function, and thus, may offer additional therapeutic targets in this axis [96].

D. Activation of Glucagon-like peptide-1 (GLP-1) and glucose-dependent insulinotropic polypeptide (GIP) receptor pathways

GLP-1 receptor agonists (GLP-1RAs; e.g., semaglutide, liraglutide, and dulaglutide), and dual GLP-1/GIP receptor agonists (e.g., tirzepatide), represent a clinically mature and promising approach to mitigate abnormal brain endothelial glycation primarily through indirect systemic effects, and potentially direct neurovascular mechanisms, including: (i) improving systemic and cellular glucose handling which reduces glycolytic overflow, (ii) enhancing Nrf2-centered antioxidant and cytoprotective pathways, thereby decreasing upstream MG generation and promoting GLO1-dependent detoxification and (iii) broader carbonyl-stress buffering [97]. Further, GLP-1RAs and dual GLP-1/GIP receptor agonists are associated with improved NO bioavailability, reduced oxidative stress, and suppression of NF-κB-mediated endothelial inflammation, all of which can attenuate AGE amplification at the endothelial luminal surface [97]. Therefore, GLP-1RAs and dual GLP-1/GIP receptor agonists may preserve brain endothelial glycocalyx architecture, indirectly through metabolic, antioxidant, and anti-inflammatory effects, offering a convergent mechanism linking metabolic intervention to brain endothelial stabilization in early preclinical AD [97]. 

Although two phase 3 trials (EVOKE and EVOKE+) of the oral GLP-1RA, semaglutide, in early symptomatic AD did not meet their primary endpoints of slowing clinical disease progression, treatment was associated with reductions in plasma high-sensitivity C-reactive protein levels, and modest exploratory reductions in several AD-relevant CSF biomarkers, including phosphorylated tau, total tau, neurogranin, and YKL-40, suggesting possible biological engagement of tau-related, inflammatory, and synaptic pathways [98,99]. Therefore, this therapeutic axis remains translationally relevant as GLP-1RAs and dual GLP-1/GIP receptor agonists may act upstream at the brain endothelial interface by reducing carbonyl stress and endothelial injury, even if downstream clinical effects were not detectable over the relatively short duration of these trials. It will be important for future studies of GLP-1 receptor-based interventions in AD to examine pharmacodynamic measures of brain endothelial injury and glycation biology as biomarkers of target engagement as well as surrogate endpoints complementary to cognitive measures [98,99]. In particular, biomarker panels incorporating carbonyl-stress markers (e.g., MG-H1 and CML/CEL), detoxification readouts (e.g., GLO1 activity), AGE-RAGE signaling markers (e.g., soluble RAGE), glycocalyx-associated markers (e.g., SDC4), and endothelial activation markers (e.g., sVCAM-1, sICAM-1, E-selectin and Ang2) will likely provide mechanistic insight into whether GLP-1 pathway engagement is effectively modulating brain endothelial pathology and assist in transitioning promising targets into Phase 3 trials that incorporate cognitive outcomes [98,99].

In silico network prioritization of GLP-1-responsive endothelial stress pathways highlights several potential complementary therapeutic nodes beyond GLP-1RAs. These include endothelial transcriptional regulators (e.g., forkhead box O1 [FOXO1] and Krüppel-like factor 2/4 [KLF2/KLF4]), metabolic-vascular regulators (e.g., sirtuin 1 [SIRT1]), membrane signaling components (e.g., caveolin-1), cAMP-dependent pathways (e.g., protein kinase A-cAMP response element-binding protein [PKA-CREB] and exchange protein directly activated by cAMP/Ras-related protein 1 [EPAC/Rap1] signaling), phosphoinositide 3-kinase–protein kinase B–endothelial NO synthase (PI3K-Akt- eNOS) signaling, AMPK, Nrf2, and the glyoxalase/redox-support network (e.g., GLO1, GLO2/HAGH, G6PD, and glutathione-dependent pathways), inflammatory convergence points (e.g., NF-κB and NOX), and glycocalyx remodeling nodes (e.g., SDC4/heparan sulfate signaling), collectively linking GLP-1/GIP receptor-responsive endothelial signaling to other axes within this framework such as carbonyl detoxification, endothelial quiescence, and endothelial glycocalyx preservation (Table 3).

E. Antidiabetic agents as potential modulators of brain endothelial glycation

In addition to metformin and GLP-1 receptor-based therapies discussed above, anti-diabetic drug classes more broadly may influence AD-relevant endothelial glycation biology by altering glucose flux and glycemic variability, MG/AGE formation and AGE-RAGE signaling, NO bioavailability, and endothelial inflammation [17,69,70,97,104].

Sodium-glucose cotransporter-2 (SGLT2) inhibitors (e.g., empagliflozin, dapagliflozin, and canagliflozin) may indirectly attenuate glycation-associated endothelial stress by promoting glucosuria and lowering systemic glycemic exposure [104,105]. SGLT2 inhibitor treatment has been associated with reduced circulating MG-H1 in individuals with type 2 diabetes [105]. Empagliflozin was associated with reduced AGE/RAGE-linked oxidative and inflammatory markers in experimental models while clinical studies suggest possible associations of SGLT2 inhibitors with lower dementia risk, although a direct specific link to brain endothelial glycation has not been established [106,107]. Dipeptidyl peptidase-4 (DPP-4) inhibitors (e.g., sitagliptin, saxagliptin, and linagliptin) also intersect with AGE-RAGE biology and endogenous incretin signaling, as DPP-4 inhibition prolongs endogenous GLP-1/GIP activity, while AGE-RAGE-induced reactive oxygen species can stimulate endothelial DPP-4 release [104,108,109]. DPP-4 inhibitors, such as linagliptin or sitagliptin, have been reported to attenuate AGE-induced RAGE, ICAM-1, plasminogen activator inhibitor-1 (PAI-1), or oxidative signaling in endothelial models [108,109].

Thiazolidinediones (e.g., pioglitazone and rosiglitazone) may influence this axis through peroxisome proliferator-activated receptor gamma (PPARγ)-mediated insulin sensitization and anti-inflammatory signaling [104,110]. Experimentally, these agents reduce RAGE expression in human endothelial cells and limit AGE- and Aβ-induced chemokine responses. However, AD prevention studies of pioglitazone have not demonstrated clear clinical efficacy, and potential safety concerns related to fluid retention and cardiac risk have limited its broad applicability [104,110,111]. Alpha-glucosidase inhibitors (e.g., acarbose and miglitol) blunt postprandial glucose increases, and thus, are relevant to the link between glycemic variability and carbonyl stress [104,112]. Acarbose has been reported to reduce serum glyceraldehyde-derived AGEs in individuals with type 2 diabetes [112]. Insulin secretagogues, including sulfonylureas (e.g., glipizide, glimepiride, and glyburide) and meglitinides/glinides (e.g., repaglinide and nateglinide), as well as insulins and insulin analogs (e.g., regular insulin, insulin lispro/aspart, insulin glargine, and insulin degludec), may reduce endothelial glycation indirectly through glucose lowering; however, their relevance to brain endothelial glycation is currently less specific and may be complicated by hypoglycemia, weight gain, and disease-stage-dependent effects on insulin signaling [104,113]. Therefore, antidiabetic agents represent metabolic interventions with variable degrees of relevance to brain endothelial glycation [17,69,70,97,104].

Future AD studies of antidiabetic agents should incorporate pharmacodynamic biomarkers of carbonyl stress, AGE burden, endothelial activation, BBB dysfunction, and brain endothelial glycocalyx injury to determine whether these therapies meaningfully modulate the proposed brain endothelial-glycation axis and whether such modulation is associated with clinical and cognitive outcomes.

**Figure 2:**
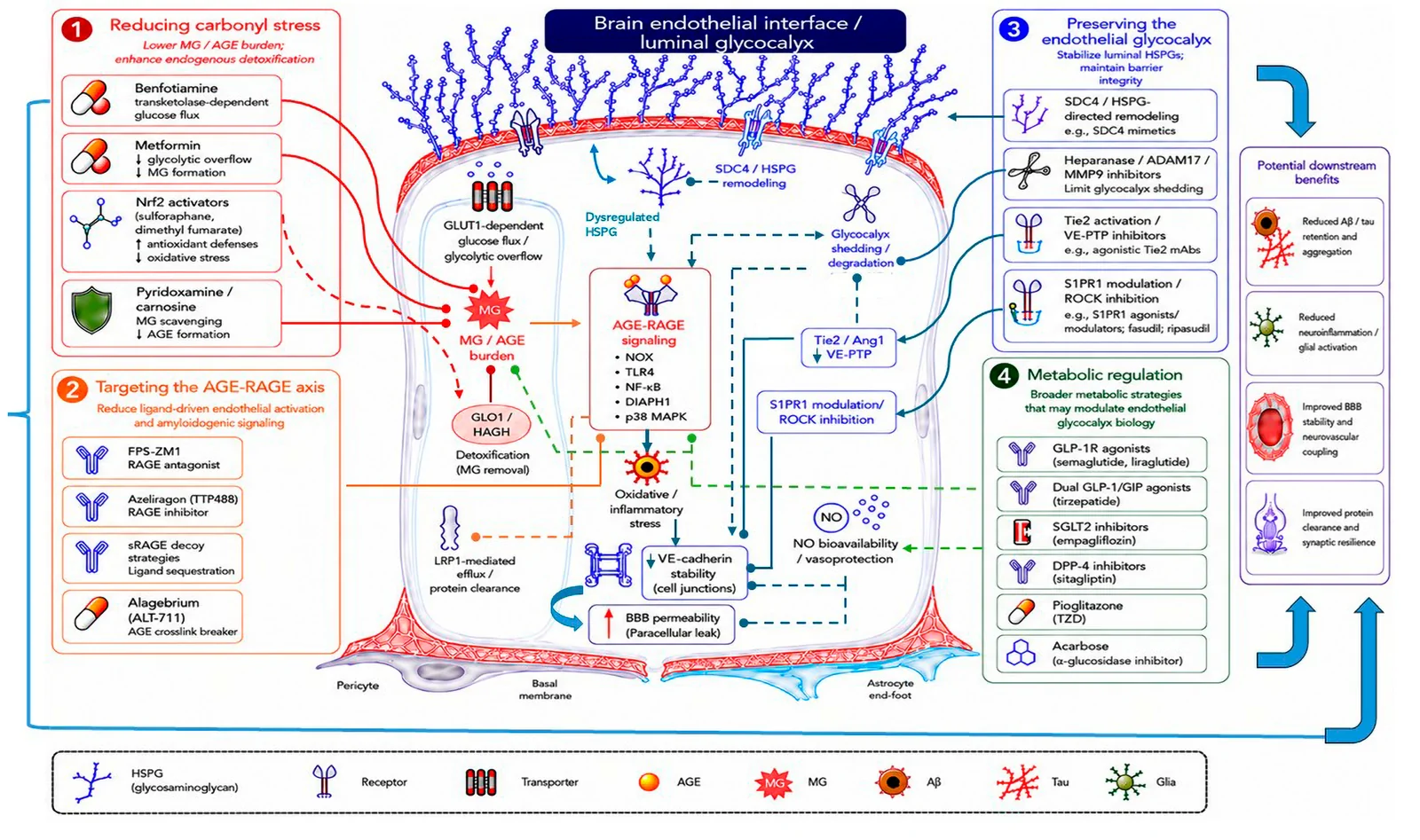
A schematic illustration of the main brain endothelial glycocalyx-related pathways that can be targeted as potential therapeutic strategies in AD. Abbreviations: Aβ, amyloid-β; AD, Alzheimer’s disease; ADAM17, a disintegrin and metalloproteinase domain-containing protein 17; AGE, advanced glycation end-product; Ang1, angiopoietin-1; BBB, blood–brain barrier; DIAPH1, diaphanous-related formin 1; DPP-4, dipeptidyl peptidase-4; GLP-1R, glucagon-like peptide-1 receptor; GLO1, glyoxalase 1; GLUT1, glucose transporter type 1; HAGH, hydroxyacylglutathione hydrolase; HPSE, heparanase; HSPG, heparan sulfate proteoglycan; LRP1, low-density lipoprotein receptor-related protein 1; MG, methylglyoxal; MMP9, matrix metalloproteinase 9; NF-κB, nuclear factor κB; NO, nitric oxide; NOX, nicotinamide adenine dinucleotide phosphate oxidase; Nrf2, nuclear factor erythroid 2–related factor 2; p38 MAPK, p38 mitogen-activated protein kinase; RAGE, receptor for advanced glycation end-products; RhoA, Ras homolog family member A; ROCK, Rho-associated coiled-coil-containing protein kinase; S1PR1, sphingosine-1-phosphate receptor 1; SDC4, syndecan-4; SGLT2, sodium-glucose cotransporter 2; sRAGE, soluble receptor for advanced glycation end-products; Tie2, angiopoietin-1 receptor/TEK receptor tyrosine kinase; TLR4, Toll-like receptor 4; **TTP488**, azeliragon; TZD, thiazolidinedione; VE-cadherin, vascular endothelial cadherin; VE-PTP, vascular endothelial protein tyrosine phosphatase. This illustration highlights major interactions and is not intended to be all-inclusive. Solid lines indicate direct or proximal relationships, dashed lines indicate indirect relationships, lines terminating in arrowheads indicate positive or stimulatory effects, and lines terminating in round ends indicate negative or inhibitory effects. This figure was generated and iteratively edited with assistance from ChatGPT (GPT-5.5 Thinking, OpenAI) using scientific content, concepts, and revision instructions provided by the author. The author reviewed the final figure and verified its scientific accuracy.

Figure 2 summarizes the main therapeutic axes which are relevant to abnormal brain endothelial glycation and offer potential targets for novel or repurposed drugs in AD.

## 4. Conclusions

We here propose a brain-endothelial glycocalyx framework for novel drug discovery and/or repurposing in AD. Specifically, we propose that brain endothelial glycation is an early event in AD pathogenesis which contributes to abnormal protein aggregation, synaptic and neuronal dysfunction and cognitive impairment. The luminal brain endothelial interface regulates the biochemical environment in which pathologic Aβ and tau accumulate, including vascular protein trafficking, inflammatory signaling, extracellular matrix composition, and neurovascular coupling. Consequently, interventions that reduce endothelial carbonyl stress and stabilize the glycocalyx architecture may limit glycation-driven modifications of aggregation-prone proteins and normalize endothelial-mediated protein transport and perivascular clearance which influence amyloid and tau burden.

**Figure 3:**
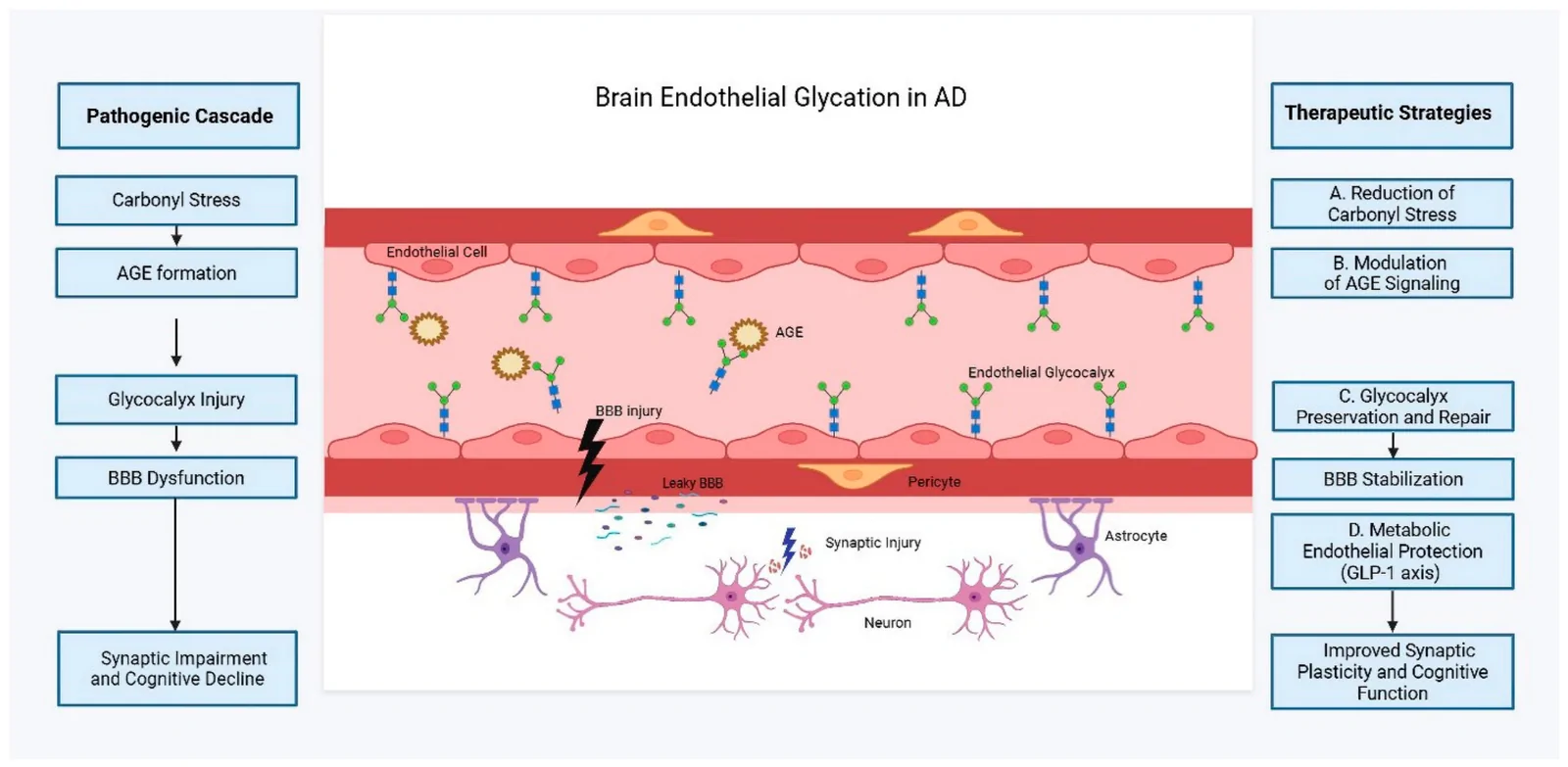
A schematic diagram representing the main therapeutic approaches proposed to target abnormal brain endothelial glycation in AD. Abbreviations: AGE, advanced glycation end-product; AD, Alzheimer’s disease; BBB, blood–brain barrier; GLP-1, glucagon-like peptide-1. This figure was created using Biorender.com.

Importantly, we propose that abnormal brain endothelial glycation has strong potential as a therapeutically actionable axis of disease modulation which may complement other therapeutic approaches that target abnormal protein aggregation. This proposed framework suggests at least four tractable intervention points: (i) reduction in upstream carbonyl stress, (ii) attenuation of AGE-RAGE signaling, (iii) preservation of the endothelial glycocalyx, and (iv) indirect endothelial protection through GLP-1 pathway modulation (Figure 3). Further, healthy brain endothelium supports neuronal survival, synaptic maintenance, and activity-dependent synaptic plasticity, including LTP, a cellular correlate of learning and memory; these processes may be disrupted by abnormal brain endothelial glycation. Therefore, we postulate that novel therapeutic strategies that target brain endothelial glycation may offer dual therapeutic benefit by downstream disease-modifying effects associated with reducing amyloid and tau aggregation as well as restoring neurovascular coupling and endothelial-neuronal/synaptic signaling pathways that can directly enhance synaptic plasticity and cognitive performance [52]. Across the broader therapeutic roadmap, agents that converge on reduced MG formation, enhanced carbonyl detoxification, attenuation of AGE-RAGE signaling, preservation of NO bioavailability, and stabilization of BBB/glycocalyx integrity may be particularly relevant to the brain endothelial glycation framework proposed herein; examples include benfotiamine, metformin, Nrf2/GLO1-targeting strategies, RAGE/sRAGE-directed approaches, Tie2/VE-PTP or S1PR1 modulation, and GLP-1-based interventions.

The immediate therapeutic implications of our current understanding of brain endothelial dysfunction as an important contributor to AD pathogenesis remain poorly investigated; partly due to presumed systemic vascular effects and the complexity of targeting the BBB. Therefore, we propose that the translational value of brain endothelial biology in AD does not lie solely in the development of novel vascular-targeted agents, but in establishing brain endothelial dysfunction as a clinically actionable axis of disease. In this framework, drug repurposing represents a practical and immediate entry point, enabling modulation of brain endothelial pathways with established safety profiles while preserving systemic vascular homeostasis. This approach shifts the focus from single-target interventions towards stabilization of the brain endothelial state and provides a foundation for precision strategies that integrate endothelial biology into the broader therapeutic landscape of AD. In this context, brain endothelial-targeting approaches are not proposed as potential alternatives to current approved therapies that target abnormal protein aggregation (e.g., FDA-approved anti-amyloid monoclonal antibodies); rather, they may offer complementary strategies to potentially enhance the efficacy of these treatments, particularly in earlier preclinical stages, or reduce the incidence of vascular side effects, the most important of which result from direct vascular injury (e.g., amyloid-related imaging abnormalities [ARIAs]).

Importantly, modulation of brain endothelial biology may prove particularly effective in primary prevention settings, where interventions are initiated before substantial amyloid and tau pathology have accumulated. Because endothelial dysfunction lies upstream of multiple pathogenic cascades in AD, including impaired protein clearance, glial dysregulation, BBB disruption, and synaptic dysfunction, targeting the brain endothelium has the potential to prevent, delay the onset, or attenuate the severity of downstream AD pathology. In this framework, brain endothelial-directed interventions may offer advantages over strategies aimed at individual pathological endpoints (e.g., those related to amyloid and tau aggregation or toxicity), by simultaneously influencing several converging drivers of disease onset and early progression and thus, are uniquely positioned as viable and promising strategies for primary prevention studies that target disease mechanisms upstream of amyloid and tau.

Brain endothelial biomarker development and updated disease models and clinical trial designs will be critical for the translation of these findings into the clinic. As novel biomarkers of brain endothelial dysfunction in AD emerge, and as brain endothelial contributions are being examined as distinct from other forms of vascular pathology in new data-driven disease models, it will be important to consider the integration of brain endothelial biomarkers into the current biomarker-based definitions and models of AD [12]. We have recently shown that CSF levels of the brain endothelial glycocalyx protein, SDC4, are increased in the earliest preclinical stages of AD and predict rates of cognitive decline and the progression of brain amyloid and tau pathologies in a large longitudinal cohort of 1041 participants who were followed over an average of 8 years [12]. Importantly, our findings suggest that CSF SDC4 predicted the progression of individuals across the different clinicopathological stages of AD (from healthy controls through the different stages of preclinical AD, leading to symptomatic AD) to a potentially better extent than other emerging markers that measure other AD pathologies such as neuronal/synaptic injury and glial dysregulation [12]. In other studies, we have also demonstrated the value of CSF VE-cadherin as a measure of brain endothelial junctional injury and its correlations with cognitive outcomes in preclinical AD [51].

Together, these findings suggest that emerging markers of brain endothelial dysfunction, including CSF SDC4 as a candidate marker of brain endothelial glycocalyx dysregulation, VE-cadherin as a candidate marker of junctional injury, biomarkers of carbonyl stress (e.g., MG-H1, CML/CEL, free MG, and GLO1 activity), and endothelial activation markers (e.g., sICAM-1, sVCAM-1, and E-selectin), may offer value as pharmacodynamic endpoints in clinical trials of disease-modifying treatments targeting diverse AD pathologies. These biomarkers may provide dynamic readouts of therapeutic effects on brain endothelial biology and help define the extent to which endothelial responses are associated with clinical and cognitive outcomes. In this context, brain endothelial biomarkers can be used to identify patient subgroups, inform disease staging, and guide therapeutic selection. Dynamic contrast-enhanced magnetic resonance imaging [MRI], arterial spin labeling, and other permeability- or perfusion-sensitive modalities may provide complementary imaging markers for brain endothelial injury. In this framework, early Phase 2 studies should implement biomarker outcomes related to carbonyl load, endothelial glycocalyx and junctional injury, and permeability domains, to detect early biological signals as a measure of target engagement, with cognitive and network-level outcomes integrated into Phase 3 studies to determine clinical efficacy.

## 5. Future Directions

Several critical directions emerge from the growing recognition of brain endothelial contributions to early AD pathogenesis, including important links between abnormal brain endothelial glycation and amyloid or tau aggregation, synaptic loss and cognitive impairment. First, future studies focusing on glycation within the cerebral microvasculature must extend past bulk tissue measurements to define the cell-type, vascular-segment, and compartment specificity of glycation-related injury. Combining single-cell and spatial transcriptomics with spatial glycoproteomics, region-specific glycome profiling, and endothelial subtype mapping will be essential to determine where carbonyl stress, glycocalyx remodeling, and altered AGE signaling first arise, which endothelial populations are most vulnerable, and how these processes evolve across the clinicopathological continuum of AD.

Second, more experimental research is needed to connect brain endothelial glycation to BBB transport mechanics and protein handling. While accumulating evidence supports effects of glycation on endothelial signaling, glycocalyx architecture, and RAGE-mediated trafficking [16,20], the impact of these changes on luminal ligand capture, caveolar and receptor-mediated transport, LRP1-dependent efflux, aggregation-prone protein retention and nucleation, and perivascular clearance remains incompletely defined. Advanced experimental platforms including human induced pluripotent stem cell (iPSC)-derived brain endothelial systems, organ-on-chip neurovascular models, and micro-perfused capillary preparations will be particularly valuable to better understand how brain endothelial glycation alters directional protein flux, BBB selectivity, local handling of aggregation-prone proteins, and endothelial-neuronal communication at high spatial and temporal resolutions.

Third, future therapeutic development should prioritize endothelial-first, mechanism-anchored strategies. Proposed therapeutic axes within this framework represent components of a multi-modality therapeutic approach. Pairing dicarbonyl-lowering or detox-enhancing therapies with interventions that reduce brain endothelial AGE sensing or stabilize glycocalyx and junctional architecture may prove especially effective, particularly because many of these targets are accessible from the luminal endothelial surface and may not require deep parenchymal penetration for biological effects. Further research is warranted to examine the translational value of the brain endothelium as a viable therapeutic target in Phase 2 and 3 clinical trials. For this approach to be successful, it will be important to identify dynamic and reliable read-outs of brain endothelial changes associated with disease onset, progression, and/or response to disease modification.

Fourth, and consistent with the above direction, the identification and validation of novel fluid or imaging markers that reliably measure brain endothelial injury, particularly markers with relative specificity for the brain rather than the systemic endothelium, and that are altered in the earliest preclinical stages of disease, will have significant implications for both clinical and research settings. Such markers would allow brain endothelial injury to be incorporated into current AD paradigms and biomarker models, including those focused on preclinical disease, while providing mechanistic insight into early pathogenesis, future disease progression, and the onset of cognitive impairment. As brain endothelial pathways are among the earliest to be dysregulated in AD, biomarkers that emerge near the time of amyloid and tau positivity and accurately reflect AD-associated brain endothelial changes, rather than nonspecific downstream consequences of neurodegeneration, could provide important incremental value beyond current predictive disease models. Further, such markers could be implemented in clinical trial selection and stratification, for example by identifying individuals with endothelial injury profiles associated with faster disease progression or higher risk from specific disease-modifying treatments. As the field moves towards spatial proteomics, transcriptomics, and multi-omics approaches for biomarker and drug target discovery, improved understanding of brain endothelial contributions to neurodegeneration may enable the identification of molecular endothelial profiles predictive of clinical and cognitive outcomes.

Together, these observations support a paradigm in which the brain endothelium is an active participant in AD onset and progression. Defining the mechanisms linking brain endothelial glycation to protein aggregation, neurovascular dysfunction, and cognitive decline may therefore uncover new opportunities for biomarker development, patient stratification, and early and more effective disease modification. By positioning the brain endothelium as a therapeutically actionable interface between systemic metabolic stress and neurodegeneration, this framework expands the current therapeutic landscape of AD and provides a roadmap for the development of complementary brain endothelium-targeted strategies in the earliest stages of disease.

## Figures and Tables

**Table 1:** Candidate therapeutic nodes in the brain endothelial carbonyl stress and AGE-RAGE pathways.

Pathway/Node	Target/Node	Mechanistic Role	Therapeutic Rationale at the BBB	Level of Evidence/Translational Status
A. Reducing Carbonyl Stress				
Carbonyl stress – MG generation control	Transketolase (TKT)	Diverts glycolytic intermediates into the pentose phosphate pathway	Reduces MG formation; targeted indirectly by benfotiamine	Human AD clinical evidence + experimental metabolic evidence. Benfotiamine has been tested in MCI/mild AD; evidence for brain endothelial glycation effects remains indirect [66,67,68].
Carbonyl stress – MG generation control	AMPK-PGC1α metabolic signaling	Regulates cellular energy balance and glycolytic flux	Shifts metabolism away from MG-producing glycolysis, lowering carbonyl stress	Human metabolic and type 2 diabetes evidence + indirect AD relevance. Metformin engages AMPK-linked pathways and is being examined in secondary AD prevention; direct evidence for modulation of brain endothelial glycation is limited [69,70,71].
Carbonyl stress – MG generation control	GLUT1/SLC2A1-dependent glucose flux	Major regulator of glucose transport across the brain endothelium	Altered endothelial glucose transport may influence local glycolytic overflow and MG generation. GLUT1 alterations have been reported in AD.	Human AD/postmortem and preclinical vascular evidence; therapeutic evidence for GLUT1 modulation in AD is indirect. Modulation of downstream GLUT1 related pathways to normalize endothelial glucose flux, glycolytic overflow, and reduce carbonyl stress, rather than direct GLUT1 inhibition, may offer therapeutic benefits in AD * [17,79,80].
Carbonyl stress – detoxification systems	Glyoxalase-1 (GLO1)	Rate-limiting enzyme detoxifying MG to D-lactate	Central endogenous defense against MG-mediated glycation	Human AD brain evidence + strong experimental evidence. GLO1 expression/activity is altered in aging and AD brains. GLO1 activation is mechanistically supported in experimental models; clinical evidence for therapeutic GLO1 modulation in AD is lacking [17,19,72,73].
Carbonyl stress – detoxification systems	Glyoxalase-2 (GLO2; HAGH)	Completes MG detoxification pathway	Supports full GLO cycle and cellular carbonyl clearance	Mechanistic/experimental evidence. Strong biochemical rationale as part of the GLO cycle; limited target-specific human AD or brain endothelial therapeutic data [17].
Carbonyl stress – detoxification systems	Aldo-keto reductases (AKR1B1, AKR1B10)	Reduce reactive carbonyl intermediates	Provide auxiliary MG detox pathways under metabolic stress	Mechanistic/experimental evidence. Relevant to carbonyl detoxification and metabolic stress biology; AD- and brain endothelial-specific therapeutic evidence remains limited [17].
Carbonyl stress – detoxification systems	Aldehyde dehydrogenases, e.g., ALDH2	Oxidize reactive aldehydes and lipid peroxidation products	Protect endothelial proteins and membranes from carbonyl damage	Preclinical vascular/endothelial evidence. ALDH2 activation, including Alda-1, has shown vascular-protective effects experimentally; clinical AD evidence is not established [17,81].
Carbonyl stress – dicarbonyl/AGE scavenging	Pyridoxamine; carnosine/anserine	Scavenge reactive carbonyl intermediates and inhibit formation of MG-derived and other AGE adducts	May reduce luminal endothelial AGE burden, carbonyl-derived glycocalyx injury, and soluble endothelial activation markers under metabolic stress	Human metabolic/translational evidence + experimental anti-glycation evidence. Pyridoxamine improved MG/AGE and endothelial activation markers in clinical studies; carnosine/anserine show experimental anti-glycation and neuroprotective effects with limited clinical cognitive benefits; direct evidence for modulation of brain endothelial glycation in AD remains limited [75,76,77,78].
Carbonyl stress – protein repair/redox buffering	DJ-1 (PARK7)	Protective protein implicated in repair or buffering of early glycation adducts	Protects proteins from MG-derived modifications	Experimental evidence; mechanism remains debated. DJ-1/PARK7 has been proposed as a deglycase or anti-glycation stress-response protein; no direct AD clinical evidence [82].
Carbonyl stress – protein repair/redox buffering	G6PD/ pentose phosphate pathway	Maintains NADPH and glutathione pools	Supports antioxidant and glyoxalase detox capacity	Mechanistic/experimental evidence with broad metabolic relevance. Supports redox buffering required for GLO activity; target-specific AD therapeutic evidence is indirect [17,83].
B. Targeting the AGE-RAGE Pathway				
AGE-RAGE signaling axis	RAGE (*AGER*)	Receptor for AGEs, HMGB1, and S100 proteins; mediates inflammatory signaling and Aβ transport	Central amplification node linking glycation to endothelial dysfunction	Human AD relevance + strong preclinical evidence + prior clinical testing. RAGE mediates Aβ transport and inflammatory signaling experimentally; the oral RAGE antagonist, azeliragon, reached clinical testing but did not establish clear disease-modifying efficacy [34,84,85,86,87].
AGE-RAGE signaling axis	Soluble RAGE (sRAGE)	Circulating decoy receptor for RAGE ligands	Sequesters AGEs and Aβ, reducing endothelial RAGE activation	Human biomarker/observational evidence + experimental rationale. sRAGE is biologically plausible as a decoy strategy, but therapeutic augmentation of sRAGE in AD remains unproven [88,89].
AGE-RAGE signaling axis	DIAPH1	Intracellular adaptor coupling RAGE to cytoskeletal and inflammatory signaling	Required for RAGE signal propagation; potential target downstream of receptor	Experimental/mechanistic evidence. Relevant as a downstream RAGE signaling node; no established human AD therapeutic evidence [84].
AGE-RAGE signaling axis	HMGB1/S100 proteins	Endogenous RAGE ligands released during stress and inflammation	Amplify AGE-RAGE signaling and neurovascular inflammation	Human inflammatory/biomarker relevance + experimental evidence. These ligands are implicated in inflammatory amplification; target-specific brain endothelial glycation therapy remains experimental [84].
AGE-RAGE signaling axis	TLR4	Cooperates with RAGE in innate immune signaling	Contributes to endothelial activation and neuroinflammatory cascades	Experimental and translational inflammatory evidence. Relevant to RAGE-associated innate immune amplification; AD brain endothelial glycation-specific therapeutic evidence is indirect [84].
AGE-RAGE signaling axis	NF-κB/p38 MAPK pathways	Major transcriptional mediators of RAGE signaling	Drive endothelial inflammation, oxidative stress, and BBB dysfunction	Strong experimental evidence; limited specificity. These pathways are well-supported inflammatory mediators but are broad, pleiotropic targets without validated AD brain endothelial glycation-directed therapy [84].
AGE crosslinks	AGE crosslink breakers, e.g., alagebrium **(ALT-711)**	Disrupt established AGE-mediated protein crosslinks	May reverse extracellular matrix and glycocalyx stiffening after AGE accumulation	Human vascular/metabolic evidence + experimental evidence; AD relevance indirect. Alagebrium has human vascular compliance/endothelial-function data, but no established AD brain endothelial therapeutic evidence [38].
AGE burden – endogenous protein repair	FN3K	Deglycation of early fructosamine adducts	Limits progression of reversible glycation products to irreversible AGEs	Mechanistic/experimental evidence. Biologically relevant to early glycation repair; human AD or brain endothelial therapeutic data is limited [90].
AGE-RAGE downstream oxidative stress	NADPH oxidase (NOX family)	Major source of RAGE-induced reactive oxygen species	Amplifies oxidative stress, endothelial activation, and BBB dysfunction downstream of AGE-RAGE signaling	Experimental vascular/neuroinflammatory evidence. Biologically plausible downstream target, but no validated AD brain endothelial glycation-specific clinical evidence [84].

The level of evidence reflects the extent to which each node is supported by human AD biomarker/clinical data, human vascular or metabolic translational evidence, preclinical endothelial/BBB evidence, or mechanistic carbonyl-stress and AGE-RAGE pathway rationale. For several targets, therapeutic relevance to AD remains indirect and is inferred from effects on MG production, AGE formation, carbonyl detoxification, redox buffering, AGE-RAGE signaling, endothelial activation, or BBB integrity rather than from direct clinical evidence in AD. * Experimental GLUT1 inhibitors have been developed as metabolic or oncological treatments; however, direct GLUT1 inhibition is unlikely to represent a desirable therapeutic strategy at the BBB as GLUT1 is required for brain glucose transport. AD-relevant strategies should aim to normalize endothelial glucose flux, glycolytic overflow, and downstream carbonyl stress rather than target GLUT1 directly. Abbreviations: Aβ, amyloid-β; AGE, advanced glycation end-product; *AGER*, gene encoding RAGE; AKR1B1/AKR1B10, aldo-keto reductase family 1 member B1/B10; ALDH2, aldehyde dehydrogenase 2; **ALT-711**, alagebrium; AD, Alzheimer’s disease; AMPK, adenosine monophosphate (AMP)-activated protein kinase; BBB, blood–brain barrier; DIAPH1, diaphanous-related formin 1; FN3K, fructosamine-3-kinase; G6PD, glucose-6-phosphate dehydrogenase; GLO, glyoxalase; GLO1, glyoxalase 1; GLO2, glyoxalase 2; GLUT1, glucose transporter type 1; HAGH, hydroxyacylglutathione hydrolase; HMGB1, high-mobility group box 1; MAPK, mitogen-activated protein kinase; MG, methylglyoxal; NADPH, reduced nicotinamide adenine dinucleotide phosphate; NF-κB, nuclear factor κB; NOX, nicotinamide adenine dinucleotide phosphate oxidase; PARK7, Parkinsonism-associated deglycase/DJ-1; PGC1α, peroxisome proliferator-activated receptor gamma coactivator 1-alpha; RAGE, receptor for advanced glycation end-products; S100, S100 calcium-binding protein family; SLC2A1, solute carrier family 2 member 1; sRAGE, soluble RAGE; TKT, transketolase; TLR4, toll-like receptor 4.

**Table 2:** Candidate therapeutic nodes regulating brain endothelial glycocalyx integrity.

Mechanistic Node	Target	Role in Glycocalyx Biology	Therapeutic Rationale in AD	Level of Evidence/Translational Status
HSPG expression and aggregation-linked remodeling	SDC4	Transmembrane HSPG regulating ligand binding, protein trafficking, mechanotransduction, and cytoskeletal organization	Upregulated in early AD; modulation of SDC4-linked heparan sulfate remodeling may reduce endothelial retention, nucleation, and impaired clearance of Aβ and tau	Human AD biomarker evidence + mechanistic HSPG/protein-aggregation rationale. CSF SDC4 is altered in early/preclinical AD and predicts amyloid, tau, and cognitive trajectories, supporting SDC4-linked glycocalyx remodeling as a potential upstream modifier of AD pathology; direct therapeutic targeting remains experimental [12,91].
Heparan sulfate biosynthesis and sulfation	EXT1/EXT2	Enzymes catalyzing heparan sulfate chain polymerization	Control structural assembly of heparan sulfate proteoglycans within the glycocalyx	Mechanistic/glycobiology evidence. Strong role in heparan sulfate biosynthesis, but limited AD-specific human or therapeutic evidence for direct targeting [96].
Heparan sulfate biosynthesis and sulfation	NDST1	N-deacetylase/N-sulfotransferase regulating heparan sulfate sulfation	Determines ligand-binding properties of glycocalyx glycosaminoglycans	Mechanistic/glycobiology evidence. Relevant to heparan sulfate sulfation and ligand binding, but AD brain endothelial therapeutic evidence is limited [96].
Heparan sulfate biosynthesis and sulfation	HS6ST1	Heparan sulfate 6-O-sulfotransferase	Modulates growth factor and chemokine interactions with the endothelial glycocalyx	Mechanistic/glycobiology evidence. Biologically plausible target for modifying glycocalyx signaling, but direct human AD or BBB-specific therapeutic evidence is not established [96].
Proteoglycan shedding and glycocalyx degradation	ADAM17	Metalloprotease mediating ectodomain shedding of syndecans and other surface proteins	Excess activity promotes glycocalyx loss and endothelial inflammation	Experimental and translational inflammatory evidence. Relevant to proteoglycan shedding and endothelial activation, but broad substrate specificity may limit direct therapeutic translation in AD [92].
Proteoglycan shedding and glycocalyx degradation	MMP9	Matrix metalloproteinase that cleaves extracellular matrix and glycocalyx components	Associated with BBB breakdown and endothelial injury in neuroinflammation	Human neurovascular/AD relevance + experimental evidence. MMP9 has been linked to BBB disruption and neuroinflammatory injury, but therapeutic inhibition in AD remains unproven and may lack target specificity [92].
Proteoglycan shedding and glycocalyx degradation	HPSE (heparanase)	Cleaves heparan sulfate chains within the glycocalyx	Drives glycocalyx thinning and promotes vascular inflammation	Experimental vascular/glycocalyx evidence; limited AD-specific translation. Heparanase is a plausible target for preserving heparan sulfate integrity, but direct human AD therapeutic evidence is limited [92].
Endothelial quiescence and glycocalyx stabilization	Tie2 receptor/TEK	Endothelial receptor maintaining vascular stability and anti-inflammatory signaling	Activation promotes glycocalyx regeneration and endothelial barrier integrity	Strong preclinical endothelial-barrier evidence + human vascular translational relevance. Tie2 signaling is a well-supported endothelial-stabilizing pathway, but AD-specific clinical evidence is not established [93,94].
Endothelial quiescence and glycocalyx stabilization	VE-PTP/PTPRB	Negative regulator of Tie2 signaling	VE-PTP inhibition activates Tie2 signaling, restoring endothelial quiescence and promoting glycocalyx recovery	Human vascular translational evidence + preclinical endothelial evidence. VE-PTP inhibition is clinically relevant in vascular disease contexts and mechanistically supports Tie2 activation, but AD brain endothelial data remain indirect [93,94].
Endothelial quiescence and glycocalyx stabilization	Angiopoietin-1 (Ang1)	Ligand activating Tie2 signaling	Supports endothelial quiescence and glycocalyx recovery	Preclinical endothelial-barrier evidence. Ang1/Tie2 activation supports endothelial stability and barrier function, but direct AD therapeutic evidence is lacking [93,94].
Endothelial quiescence and glycocalyx stabilization	Angiopoietin-2 (Ang2)	Context-dependent antagonist of Tie2	Elevated Ang2 destabilizes endothelium and promotes glycocalyx loss	Human vascular biomarker/translational evidence + experimental evidence. Ang2 is a clinically relevant marker and mediator of endothelial destabilization, but AD-specific therapeutic modulation remains indirect [93,94].
Endothelial quiescence and glycocalyx stabilization	S1PR1	Promotes VE-cadherin retention, cortical actin organization, and endothelial barrier stability	Counteracts inflammatory and glycation-induced junctional destabilization and supports glycocalyx recovery	Human clinical class relevance + preclinical endothelial-barrier evidence. S1P receptor modulation is clinically established in immune/CNS disease contexts, and S1PR1 supports barrier stabilization experimentally, but AD brain endothelial glycation-specific evidence is limited [95].
Cytoskeletal barrier regulation	RhoA/ROCK signaling	Controls endothelial contractility and junctional tension	Excess activation promotes VE-cadherin internalization and BBB dysfunction during glycocalyx injury	Preclinical endothelial/BBB evidence + limited human vascular translational relevance. ROCK inhibition is biologically plausible for reducing endothelial contractility and barrier dysfunction, but AD-specific clinical evidence is not established [15,60,95].

The level of evidence reflects the extent to which each node is supported by human AD biomarker/clinical data, human vascular or translational evidence, preclinical endothelial/BBB evidence, or mechanistic glycobiology rationale. For several targets, therapeutic relevance to AD remains indirect and is inferred from effects on endothelial barrier stability, glycocalyx preservation, vascular inflammation, or BBB integrity rather than from direct clinical evidence in AD. Abbreviations: AD, Alzheimer’s disease; ADAM17, a disintegrin and metalloproteinase domain-containing protein 17; Ang1, angiopoietin-1; Ang2, angiopoietin-2; BBB, blood–brain barrier; CNS, central nervous system; EXT1/EXT2, exostosin glycosyltransferase 1/2; HPSE, heparanase; HSPG, heparan sulfate proteoglycan; HS6ST1, heparan sulfate 6-O-sulfotransferase 1; MMP9, matrix metalloproteinase 9; NDST1, N-deacetylase/N-sulfotransferase 1; PTPRB, protein tyrosine phosphatase receptor type B; RhoA, Ras homolog family member A; ROCK, Rho-associated coiled-coil-containing protein kinase; S1PR1, sphingosine-1-phosphate receptor 1; SDC4, syndecan-4; TEK/Tie2, TEK receptor tyrosine kinase/angiopoietin-1 receptor; VE-cadherin, vascular endothelial cadherin; VE-PTP, vascular endothelial protein tyrosine phosphatase.

**Table 3:** Proposed endothelial signaling nodes linking GLP-1 receptor signaling to reduced carbonyl stress and BBB stabilization.

Signaling Node	Target/Pathway	Mechanistic Role in Brain Endothelium	Relevance to Glycation and BBB Stability	Level of Evidence/Translational Status
GLP-1R signaling	GLP-1R	G-protein-coupled receptor activating cAMP-dependent protective signaling in an endothelial and neurovascular context	Primary pharmacologic entry point for GLP-1RAs; activates GLP-1R- dependent signaling relevant to endothelial metabolic resilience and BBB/glycocalyx stability	Human clinical evidence in metabolic disease + AD clinical trial evidence; brain endothelial glycation evidence indirect. GLP-1RAs are clinically established for type 2 diabetes and obesity and have been tested in AD. Direct evidence that either class reduces brain endothelial glycation remains limited [97,98,99].
cAMP signaling cascade	PKA/CREB axis	Transcriptional activation of metabolic and cytoprotective pathways	Promotes endothelial resilience and suppresses inflammatory gene expression	Mechanistic/preclinical evidence. This is a canonical downstream GLP-1 signaling pathway; target-specific human AD or brain endothelial glycation evidence is not established [97].
cAMP-dependent barrier signaling	EPAC1/Rap1 axis	cAMP effector pathway that stabilizes endothelial junctions, promotes VE-cadherin retention	Provides a direct mechanistic bridge between GLP-1R/cAMP activation and BBB stabilization	Preclinical endothelial-barrier evidence; AD brain endothelial glycation-specific evidence remains indirect [97,100].
Nitric oxide (NO) signaling	PI3K-Akt-eNOS pathway	Enhances NO production and vascular relaxation	Restores endothelial function, improves neurovascular coupling, and reduces oxidative stress	Human vascular relevance + strong experimental endothelial evidence. This pathway is central to endothelial function and NO bioavailability; evidence for direct modulation of AD brain endothelial glycation is limited [49,55,97].
Metabolic stress sensing	AMPK signaling	Coordinates cellular energy balance and reduces glycolytic overflow	May lower MG generation and carbonyl stress	Human metabolic evidence + experimental evidence; AD relevance indirect. AMPK-linked interventions such as metformin are clinically established and being examined in secondary AD prevention; direct brain endothelial glycation effects are not established [69,70,71,97].
Antioxidant transcriptional pathways	Nrf2 pathway	Master regulator of cellular detoxification and antioxidant defenses	Enhances GLO1 activity and protects against MG-induced endothelial injury	Preclinical evidence + human translational relevance. Nrf2 activation has mechanistic support for carbonyl/redox stress buffering; clinically used Nrf2 activators provide translational relevance; AD brain endothelial glycation evidence remains indirect [72,73,74,97].
Carbonyl detoxification network	GLO1/GLO2 (HAGH)	Glyoxalase system detoxifying MG	Limits AGE formation on endothelial proteins	Human AD brain evidence for GLO1 + strong mechanistic evidence; therapeutic evidence limited. GLO1 dysfunction has been described in AD brains, and the glyoxalase system is central to MG detoxification; clinical evidence for therapeutic modulation in AD is lacking [17,19,72,73].
Carbonyl detoxification network	G6PD/pentose phosphate pathway	Maintains NADPH and glutathione pools	Supports redox buffering required for glyoxalase activity	Mechanistic/metabolic evidence. Relevant to NADPH, glutathione, and glyoxalase support; direct human AD or brain endothelial therapeutic evidence is limited [17,83].
Inflammatory signaling suppression	NF-κB pathway	Central regulator of endothelial inflammatory responses	GLP-1 signaling suppresses NF-κB activation and endothelial inflammation	Strong experimental evidence; broad target with limited specificity. NF-κB is highly relevant to endothelial inflammation and AGE-RAGE signaling; however, it is pleiotropic and not a specific brain endothelial glycation therapeutic target [84,97].
Oxidative stress amplification	NADPH oxidase/ NOX family	Major endothelial source of reactive oxygen species	GLP-1 signaling may reduce NOX-driven oxidative stress	Experimental vascular/endothelial evidence. NOX is a plausible downstream oxidative-stress target; however, AD brain endothelial glycation-specific clinical evidence is not established [84,97].
Endothelial stress transcription	FOXO1	Regulates endothelial metabolism and junctional stability	GLP-1-Akt signaling may suppress FOXO1-driven endothelial stress	Preclinical/mechanistic evidence. FOXO1 is relevant to endothelial metabolic stress and downstream Akt signaling; AD-specific or brain endothelial glycation-directed therapeutic evidence is limited [101].
Endothelial homeostasis transcription	KLF2/KLF4	Promotes anti-inflammatory endothelial phenotype and eNOS expression	May link GLP-1 cAMP signaling to endothelial quiescence and glycocalyx stability	Preclinical endothelial-homeostasis evidence. KLF2/KLF4 are well-supported endothelial quiescence factors; direct human AD or brain endothelial glycation therapeutic evidence is not established [101].
Metabolic-vascular integration	SIRT1	Regulates oxidative stress, mitochondrial function, and inflammation	Potential mediator of GLP-1 vascular-protective effects	Experimental and broad translational metabolic evidence. SIRT1 is relevant to metabolic-vascular resilience, oxidative stress, and inflammation; however, its role as a GLP-1-linked brain endothelial glycation target in AD remains indirect [102].
Glycocalyx remodeling control	SDC4/heparan sulfate signaling	Structural component of the brain endothelial glycocalyx; HSPG scaffold regulating glycocalyx architecture, ligand binding, and mechano- transduction	May serve as a downstream readout or effector of GLP-1R-linked endothelial stabilization; direct modulation remains unproven	Human AD biomarker evidence + mechanistic glycocalyx evidence; therapeutic evidence indirect. SDC4 is altered in early AD and relevant to glycocalyx injury; however, evidence that GLP-1R activation normalizes SDC4 or brain endothelial glycation in AD is not established [12].
Caveolar transport and membrane signaling	CAV1	Controls caveolae-mediated signaling and endothelial transport	May influence BBB transcytosis, endothelial transport and response to glycation-related stress	Preclinical BBB/endothelial transport evidence. CAV1 is mechanistically relevant to endothelial transcytosis and BBB signaling; however, direct AD therapeutic evidence for targeting CAV1-mediated glycation responses is limited [103].

The level of evidence reflects the extent to which each signaling node is supported by human clinical or biomarker data, human vascular/metabolic translational evidence, preclinical endothelial/BBB evidence, or mechanistic pathway rationale. Abbreviations: AGE, advanced glycation end-product; Akt, protein kinase B; AD, Alzheimer’s disease; AMPK, adenosine monophosphate (AMP)-activated protein kinase; BBB, blood–brain barrier; cAMP, cyclic adenosine monophosphate; CAV1, caveolin-1; CREB, cAMP response element-binding protein; eNOS, endothelial nitric oxide synthase; EPAC/Rap1, exchange protein directly activated by cAMP/Ras-related protein 1; FOXO1, forkhead box O1; G6PD, glucose-6-phosphate dehydrogenase; GLO1, glyoxalase 1; GLO2, glyoxalase 2; GLP-1, glucagon-like peptide-1; GLP-1R, glucagon-like peptide-1 receptor; HAGH, hydroxyacylglutathione hydrolase; KLF2/KLF4, Krüppel-like factor 2/4; MG, methylglyoxal; NADPH, reduced nicotinamide adenine dinucleotide phosphate; NF-κB, nuclear factor κB; NO, nitric oxide; NOX, nicotinamide adenine dinucleotide phosphate oxidase; Nrf2, nuclear factor erythroid 2–related factor 2; PI3K, phosphoinositide 3-kinase; PKA, protein kinase A; SDC4, syndecan-4; SIRT1, sirtuin 1.

## Data Availability

No new data were created or analyzed in this study. Data sharing is not applicable to this article.
